# Human nasal septal chondrocytes (NSCs) preconditioned on NSC-derived matrix improve their chondrogenic potential

**DOI:** 10.1186/s40824-021-00211-z

**Published:** 2021-04-06

**Authors:** Yong Kwan Noh, Sung Won Kim, Ik-Hwan Kim, Kwideok Park

**Affiliations:** 1 Center for Biomaterials, Korea Institute of Science and Technology (KIST), 02792 Seoul, Republic of Korea; 2 Department of Biotechnology, Korea University, 02841 Seoul, Republic of Korea; 3 Department of Otolaryngology-Head and Neck Surgery, College of Medicine, The Catholic University of Korea, 06591 Seoul, Republic of Korea; 4 Division of Bio-Medical Science and Technology, KIST School, University of Science and Technology (UST), 02792 Seoul, Republic of Korea

**Keywords:** Cartilage, Extracellular matrix (ECM), Human nasal septal chondrocyte (NSC), Chondrocyte‐derived extracellular matrix, Preconditioning

## Abstract

**Background:**

Extracellular matrix (ECM) has a profound effect on cell behaviors. In this study, we prepare a decellularized human nasal septal chondrocyte (NSC)-derived ECM (CHDM), as a natural (N-CHDM) or soluble form (S-CHDM), and investigate their impact on NSCs differentiation.

**Methods:**

N-CHDM, S-CHDM were obtained from NSC. To evaluate function of NSC cultured on each substrate, gene expression using chondrogenic marker, and chondrogenic protein expression were tested. Preconditioned NSCs-loaded scaffolds were transplanted in nude mice for 3 weeks and analyzed.

**Results:**

When cultivated on each substrate, NSCs exhibited similar cell spread area but showed distinct morphology on N-CHDM with significantly lower cell circularity. They were highly proliferative on N-CHDM than S-CHDM and tissue culture plastic (TCP), and showed more improved cell differentiation, as assessed via chondrogenic marker (Col2, Sox9, and Aggrecan) expression and immunofluorescence of COL II. We also investigated the effect of NSCs preconditioning on three different 2D substrates while NSCs were isolated from those substrates, subsequently transferred to 3D mesh scaffold, then cultivated them *in vitro* or transplanted *in vivo*. The number of cells in the scaffolds was similar to each other at 5 days but cell differentiation was notably better with NSCs preconditioned on N-CHDM, as assessed via real-time q-PCR, Western blot, and immunofluorescence. Moreover, when those NSCs-loaded polymer scaffolds were transplanted subcutaneously in nude mice for 3 weeks and analyzed, the NSCs preconditioned on the N-CHDM showed significantly advanced cell retention in the scaffold, more cells with a chondrocyte lacunae structure, and larger production of cartilage ECM (COL II, glycosaminoglycan).

**Conclusions:**

Taken together, a natural form of decellularized ECM, N-CHDM would present an advanced chondrogenic potential over a reformulated ECM (S-CHDM) or TCP substrate, suggesting that N-CHDM may hold more diverse signaling cues, not just limited to ECM component.

## Background

Articular cartilage in knee is a typical loading-bearing tissue where only single cell type, chondrocyte exists [1]. The major functions of chondrocyte include cartilage-specific extracellular matrix (ECM) production, tissue maintenance and remodeling that are essential for cartilage homeostasis [2]. Once damaged, however articular cartilage commonly experiences a gradual tissue degeneration and loss that barely self-heals, due mainly to the avascularity and low cellularity in the cartilage tissue [3]. Many surgical interventions including microfracture in clinics have been developed and proven some improvement but they still fall short of a functional cartilage recovery [4, 5].

Therefore, cartilage regeneration has been a very active and promising research topic since tissue engineering emerged in the early 1990 s [6]. Among the major components of conventional tissue engineering (cell, scaffold, growth factor), cell source is a critical part, because cells are the key player for initiation, progression, and maturation of target tissue [7]. In this regard, mesenchymal stem cells (MSCs) and primary chondrocytes have been the two primary cell source of cartilage regeneration [8]. MSCs are commonly harvested from bone marrow, fat, or umbilical cord blood [9]. It is a general practice of stem cell therapy that MSCs are largely expanded *in vitro* and transplanted into cartilage defect site with or without carrier material [10]. Meanwhile autologous or allogenic chondrocytes can be harvested from knee articular cartilage, nasal septum, or costal cartilage [11]. Those primary chondrocytes are generally cultivated on tissue culture plastic in a serum condition and undergo multiple passages to obtain a large cell number [12]. During such cell passage, a critical point is that chondrocytes experience cell dedifferentiation, a phenomenon of losing cell phenotype and eventually turning to fibroblastic cells [13]. To delay this shift or to trigger cell redifferentiation, there have been quite a few studies that include three-dimensional (3D) culture environment, substrate coating or modifications [14]. Among them, 3D culture in hydrogel and substrate coating using collagen or fibronectin are the most common practices [15].

Previously we have investigated ECM, specifically cell-derived ECM (CDM) as a new source of biomimetic material toward cell expansion and tissue regeneration. The CDM is obtained after the clearance of the cellular components of cultivated cells *in vitro* via decellularization [16]. Our early works have shown a great potential in cartilage regeneration as well as in MSCs differentiation into chondrogenic lineage. In fact, ECM is known to have numerous functions *in vivo*: cell adhesion, cell migration, storage of signaling molecules, and external signal transmittance to cells, among the listed ones [17]. ECM provides cells with specific microenvironments so that they would maintain cell phenotype and eventually perform normal tissue/ organ function in the body. Numerous documents have demonstrated the benefits of ECM or individual ECM components toward regenerative purpose [18]. The major advantages of such ECM are generally attributed to compositional homology and/or mechanical compliance to a target tissue, while ECM *in vitro* is expected to mimic *in vivo* cellular microenvironment.

In this study, we prepare nasal septal chondrocytes (NSCs)-derived ECM (CHDM) and examine its impact on NSCs behaviors, such as cell attachment, proliferation, and differentiation. As a promising source of primary chondrocytes, NSCs are isolated from nasal septal tissues that are surgically harvested from patients undergoing septoplasty [19]. Compared to the chondrocytes from knee articular cartilage, NSCs are beginning to find applications in cartilage regeneration [20]. Our hypothesis is that NSCs-derived matrix may provide a natural ECM environment to NSCs and thus should be beneficial in the maintenance of NSC phenotype and cell differentiation. We prepare two different types of CHDM, natural and soluble CHDM in an attempt to compare the difference between naturally assembled ECM structure (N-CHDM) and reformulated one (S-CHDM) on NSCs responses. NSCs are allowed to grow and adapt on two different ECM substrates or TCP (a control). We call this process a preconditioning of NSCs while they interact with different substrate microenvironments. Furthermore when those preconditioned cells on 2D substrate are transferred into 3D environment, their chondrogenic potency is evaluated *in vitro* and using *in vivo* ectopic model. Interestingly, the NSCs preconditioned on the N-CHDM showed significantly better chondrogenic activity as assessed via chondrocyte phenotype (lacunae) and newly synthesized ECM than those on S-CHDM or TCP. Current study suggests that given ECM microenvironment poses a significant impact on the NSCs differentiation, with the preference on naturally assembled ECM architecture.

## Materials and methods

### Nasal septal chondrocytes (NSCs) isolation

All the studies using nasal septal chondrocytes (NSCs) were conducted after written approval (HC13TISI0038) obtained from the Institutional Review Board of the Catholic Medical Center Clinical Research Coordinating Center. Human nasal septum tissue was harvested from five patients who were undergoing septoplasty [21]. Those tissues were cut into 1 mm^3^ pieces and they were enzymatically digested using 0.2 % protease solution (Gibco) for 60 min, followed by incubation in 0.3 % collagenase (Sigma-Aldrich) for 12 h at 37 °C. The isolated cells were then seeded in a 75-mm^2^ cell culture flask (NUNC) and cultivated in low-glucose Dulbecco’s Modified Eagle Medium (Gibco-BRL) with 10 % fetal bovine serum (FBS; Gibco) and antibiotics at 37 °C in 5 % CO_2_ incubator. The confluent chondrocytes were subcultured following a standard protocol using 0.05 % trypsin/EDTA solution (Gibco).

### Preparation of NSCs-derived matrix: N-CHDM and S-CHDM

NSCs are loaded at the density of 1.3 × 10^4^ cells/cm^2^ in a 100 mm diameter petri-dish and cultured for 7 days. At the time of confluence, the culture plates were washed twice with phosphate buffered saline (PBS) and subsequently subject to decellularization process. They were incubated briefly in a detergent solution containing 0.15 % Triton X-100 and 10 mM NH_4_OH (Sigma-Aldrich) at 37 °C, then treated with 50 U/mL DNase I and 2.5 µL/mL RNase A (Invitrogen) for 1 h, finally washed with PBS thoroughly and stored at 4 °C before use. We name it a natural NSC-derived matrix (N-CHDM). Meanwhile after the decellularization, N-CHDM was collected by gentle pipetting and this was transferred to 1 ml EP tube, then digested using 1 mg/ml pepsin (Sigma-Aldrich) in 0.01 N HCl for 48 h at 37 °C. These digested N-CHDM was neutralized via 0.1 N NaOH, and it was then diluted with 1X PBS. We call this a soluble NSC-derived matrix (S-CHDM). To prepare an S-CHDM substrate, the solution (50 µg/ml) was loaded onto TCP and incubated at 37 °C for 1 h as similar to fibronectin coating.

### Characterization of N-CHDM

The surface morphology of N-CHDM was observed using phase contrast microscope (Carl Zeiss) and scanning electron microscope (SEM; Phenom G2 Pro Desktop). For immunofluorescence staining of ECM components, N-CHDM was prepared on the glass coverslips, fixed in 4 % paraformaldehyde, then washed with PBS more than three times. Subsequently these samples were treated with 0.2 % Triton X-100 solution for 30 min and they were blocked with 3 % bovine serum albumin (BSA). Once primary antibodies against human fibronectin (Santa Cruz, sc-271,098) and type 2 collagen (COL II) (Abcam, ab34712) were prepared in 1 % bovine serum albumin (BSA) solution, while diluted in 1:200, they were separately treated overnight at 4^o^C. As the secondary antibodies diluted in 1:500, Alexa fluoro-488 was used for fibronectin and rhodamine red-X (Invitrogen) was applied for COL II. The intensity and distribution of these ECM proteins were confirmed via confocal microscope (LSM700; Carl Zeiss).

### NSC viability and proliferation

NSCs (P5) were seeded on three different substrates: tissue culture plate (TCP), S-CHDM, and N-CHDM, respectively. After 24 h culture on each substrate, cell viability was assessed via LIVE/DEAD® Viability/Cytotoxicity Kit (Invitrogen). Live or dead cells are visualized in green and red, respectively using a fluorescent microscope. Evaluation of cell proliferation was also carried out at 48 and 96 h using Cell Counting kit-8 (CCK-8; Dojindo). Briefly, each sample was added with 10 % CCK-8 solution and incubated at 37 °C for 2 h. The supernatant (200 µL) was then transferred to a 96-well plate and the absorbance of each sample was measured at 450 nm using a Multiskan microplate reader.

### Focal adhesion (FA) assessment

Cell morphology on TCP, S-CHDM, and N-CHDM was also analyzed in 24 h. For this, NSCs were fixed with 4 % paraformaldehyde for 30 min, gently washed with PBS, and permeabilized with 0.2 % Triton X-100 for 20 min, then blocked with 1 % BSA for 1 h. Each sample was incubated with primary antibody against vinculin (Santa Cruz, sc-73,614) in 1 % BSA (1:300) overnight at 4 °C. After being rinsed three times with PBS, they were incubated with Alexa-Fluor-488-conjugated goat anti-mouse IgG (Invitrogen, A11001) in 1 % BSA (1:200) for 1 h at room temperature in the dark, followed by incubation with rhodamine phalloidin (Invitrogen, R415) for 30 min. Once these samples were washed, they were then mounted and observed via confocal microscope (LSM700; Carl Zeiss). Cell morphology was quantitatively assessed via cell spreading area and cell circularity. Those data were obtained by manually outlining cell borders (10 cells) per sample (n = 5, each group) and by processing the raw data using ImageJ software. In addition, the mean area of FA was also quantified for each test group using ImageJ.

### Immunofluorescence staining of COL II

NSCs differentiation on three different microenvironments was carried out under the 10 % serum condition for 7 days and the result was assessed via immunofluorescence of COL II. The NSCs-loaded substrate was washed twice with PBS and blocked for 45 min with 3 % BSA. After they were incubated overnight with a mouse monoclonal anti-Col II (Abcam, ab34712) (1:200) at 4 °C, the samples were washed three times with PBS, incubated for 1 h with Alexa Fluor 488 goat anti-mouse IgG (1:200) at room temperature, and then rinsed with PBS. At the same time, DAPI staining was also conducted for nucleic detection. The fluorescent signals of COL II were detected using confocal microscope (LSM700; Carl Zeiss).

### Quantitative real-time polymerase chain reaction (q-PCR)

For gene expression level of chondrogenic markers, q-PCR was carried out using the NSCs grown on three different substrates, respectively. First of all, total RNA was isolated from the NSCs of each group (n = 3) using TRIzol RNA Isolation Reagents (Invitrogen). The single-stranded cDNA was then prepared via a solution of RNA extracts, primers and reverse transcription (RT) reaction mixture. The reaction product (1 µL) was mixed with Maxime PCR PreMix (Intron) and Taqman primers and probes, then followed by polymerase chain reaction via Applied Biosystems 7300 Real-Time PCR system. The relative gene expression was calculated using ΔΔCt method, where each sample was internally normalized to glyceraldehyde-3-phosphate dehydrogenase (GAPDH). The chondrogenic markers tested in this study are SRYbox containing gene 9 (Sox 9), Col 2, Aggrecan, and Col 10. Target genes and their primers are as follows: Sox9: AAAGGCAAGCAAAGGAGATG (forward) and TGGTGTTCTGAGAGGCACAG (reverse); Col 2: AAGGCTCCCAGAACATCACC (forward) and ATCCTTCAGGGCAGTGTACG (reverse); Aggrecan: TCTGTAACCCAGGCTCCAAC (forward) and TGGAGTACCTGGTGGCTCTC (reverse); Col 10: TGGGACCCCTCTTGTTAGTG (forward) and TTGGGTCATAATGCTGTTGC (reverse); housekeeping gene (GAPDH): GGGCTCTCCAGAACATCATC (forward) and TTCTAGACGGCAGGTCAGGT (reverse). The raw data are first normalized to GAPDH, and then normalized to that of TCP sample at 0 day. The results of gene expression are shown as a fold change relative to that of TCP (0 day).

### Western blot analysis

For further analysis of chondrogenic markers, the NSCs were lysed using RIPA lysis buffer (Sigma-Aldrich). A total of 30 µg protein was subject to SDS-polyacrylamide gel electrophoresis (SDS-PAGE) with the use of 10 % resolving gels, then followed by transfer to polyvinylidene fluoride (PVDF) membrane (Bio-Rad). The membrane was incubated with primary antibodies specific for COL II (Abcam, ab34712), SOX9 (Santa Cruz, sc-166,505), Aggrecan (Santa Cruz, sc-70,332), COL I (Abcam, ab34710), and β-actin (Santa Cruz, sc-47,778), respectively, all of which were diluted in 1:1000. Those markers were visualized by chemiluminescence using Western ECL substrate (Thermo Scientific), and the luminescent images were analyzed using a LAS-3000 (Fujifilm).

### NSCs differentiation in 3D polymer mesh scaffold***in vitro***

To investigate the effect of NSCs grown on different 2D substrates, we transferred the NSCs into a 3D environment and examined the NSCs differentiation for up to 3 weeks. Poly l-lactic acid /polypropylene (PLLA/PP) (Ingelheim, Germany) microfibers were prepared using a rotary cutter and their nonwovens were fabricated into a mesh scaffold via modified wet-laid process as previously reported [22]. The surface morphology of mesh scaffold was examined via the SEM. Before cell seeding (1 × 10^5^), mesh scaffolds were coated with fibronectin to ensure good cell attachment. After 24 h post-seeding, cell attachment in the scaffolds were evaluated via DAPI staining. The NSCs proliferation was also assessed at day 1, 3 and 5, respectively using CCK-8 assay. In addition, NSC differentiation in the scaffolds was assessed at 3 weeks via immunofluorescence of COL II, along with F-actin and DAPI staining, respectively. The fluorescent signals were detected using confocal microscope (LSM700; Carl Zeiss). The test samples are triplicated for each group.

### Transplantation of NSCs-loaded mesh scaffold into ectopic model***in vivo***

Animal study was implemented according to the guideline of the Institutional Animal Care and Use Committee of the Korea Institute of Science and Technology (IACUC, 2018-003). A subcutaneous ectopic model was prepared using 4 week-old SCID mice (Narabiotech, Korea). The experimental groups were divided into three (n = 4, each group), based on the NSCs that were pre-conditioned on TCP, S-CHDM, and N-CHDM, respectively. Prior to the *in vivo* transplantation, PKH-26 (PKH26 Red Fluorescent Cell Linker Kit, Sigma-Aldrich) labelled NSCs were prepared and they were then seeded at 5 × 10^5^ cells per mesh scaffold. After 3 days of incubation *in vitro*, they were subcutaneously transplanted into the back of the mice right after anesthesia with Avertin. All the animals were sacrificed via overdose of anesthesia at 3 weeks post-transplantation. For histological analysis, we harvested the skin flaps at the transplantation site of each animal and processed them.

### Histology: Hematoxylin-eosin and Safranin O staining

After the transplants were retrieved, each mesh scaffold was fixed in 4 % paraformaldehyde solution for 3 h, dehydrated with 100 % ethanol, washed with xylene, and then embedded in paraffin block. Thin sections of 5 μm thickness were made and placed on the glass slide. Once the specimens were deparaffinized using xylene and ethanol, they were stained by hematoxylin, followed by counterstaining of eosin. For quantitative analysis, PKH-26 labelled NSCs were observed via confocal microscope and the positive signals were analyzed quantitatively, based on the ratio of the positive area to the total area of the representative images (n = 5, per sample; 4 samples, each group) using imageJ. The number of chondrocyte with a lacunae structure was also calculated, based on the average count of NSCs with lacunae in the given area using imageJ (n = 5, per sample). Each sample is triplicated for each group. In addition, a routine protocol of Safranin-O/fast green staining was applied to the sections using aqueous Safranin-O (0.1 %, w/v) and fast green solution (0.001 %, w/v). Histological images were photographed using an optical microscope (Carl Zeiss).

### Statistical analysis

All the data are expressed as mean ± standard deviation. Statistically significant difference is sought via one-way analysis of variance (ANOVA) with a post-hoc, Bonferroni’s multiple comparison tests using GraphPad Prism 7 (GraphPad Software). A significant difference is marked as * (*p* < 0.05), ** (*p* < 0.01), or *** (*p* < 0.001).

## Results

### Characterization of N-CHDM and S-CHDM 

**Fig. 1:**
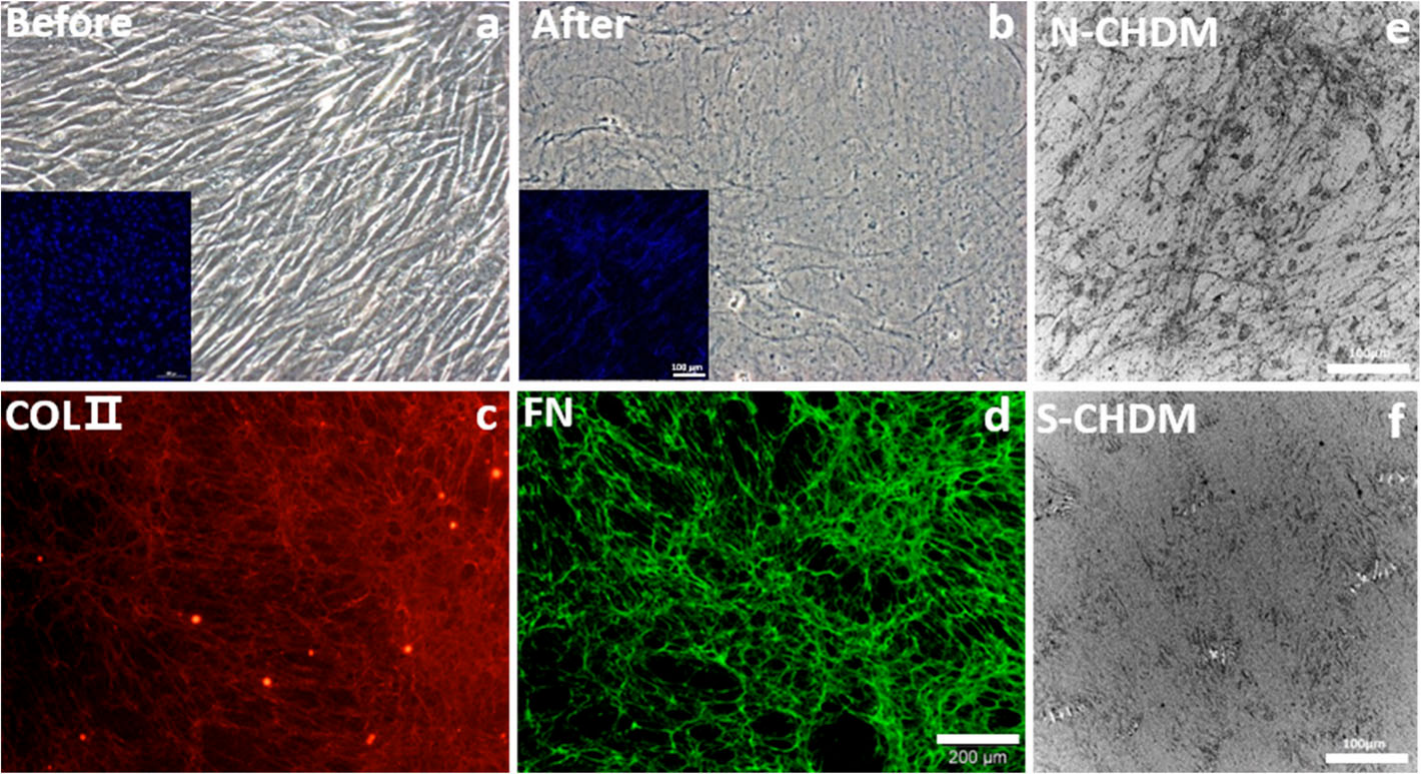
Preparation of NSC-derived ECM and characterization. Human nasal septal chondrocyte derived extracellular matrix (N-CHDM) is obtained from decellularization of *in vitro* cultured nasal septal chondrocytes on TCP. Cell morphology (**a**) before and (**b**) after decellularization. The inset images are DAPI staining that demonstrates the clearance of cell nucleic components after decellularization. The identity of ECM macromolecules, **c** type 2 collagen (COL II) and (**d**) fibronectin (FN), as assessed via immunofluorescence staining. The scale bar is 200 μm. Observation of the surface morphology of (**e**) N-CHDM and (**f**) S-CHDM via SEM (The scale bar is 100 μm)

NSCs (passage 5) are confluent during 7 days of culture (Fig. 1a). After the decellularization, we noticed that a unique morphology of N-CHDM with a fibrous structure was covered over the surface area (Fig. 1b). The inset images are DAPI staining that demonstrates the clearance of nucleic components after decellularization. In addition, the identity of ECM components within the N-CHDM like COL II (Fig. 1c) and fibronectin (FN) (Fig. 1d) was confirmed via immunofluorescence staining. When the surface morphology of N-CHDM (Fig. 1e) and S-CHDM (Fig. 1f) was observed via SEM, the difference was clear between them, due to the fibers network formed on N-CHDM only.

### NSCs adhesion, proliferation, and differentiation

**Fig. 2:**
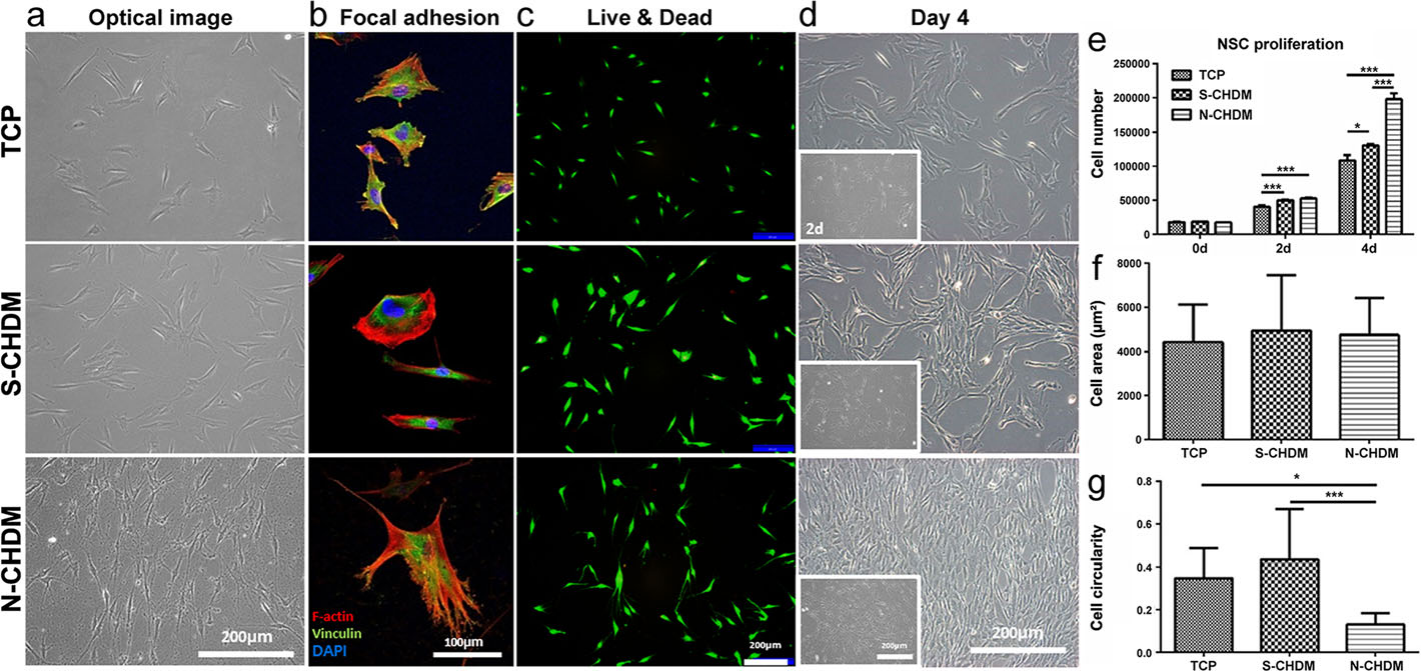
NSC morphology, focal adhesion and proliferation on each substrate. NSCs (P5) seeding on three different substrates (TCP, S-CHDM, N-CHDM) and observation of (**a**) cell morphology, **b** focal adhesion (F-actin: red, DAPI: blue, vinculin: green) and **c** Live&Dead staining (live: green, dead: red) for cell viability on each substrate on day 1. **d** NSC growth as monitored at 2 (inset) and 4 days. **e** Cell proliferation of NSCs at 0, 2, and 4 days as evaluated via CCK-8 assay (*n* = 3, each group). Quantitative analysis of (**f**) cell area and (**g**) cell circularity. Statistically significant difference as marked *p < 0.05 or ***p < 0.001

NSCs adhesion on three different substrates (TCP, S-CHDM and N-CHDM) was observed using optical microscopy (Fig. 2a). Upon focal adhesion staining, we noticed more spreading cell morphology on the ECM substrate, especially on the N-CHDM (Fig. 2b). The live and dead staining confirms no cytotoxicity of both ECM types (Fig. 2c). As for cell growth rate, it was obvious that NSCs on the N-CHDM was greater on cell proliferation than those on the other substrates at 4 days (Fig. 2d). The difference was statistically significant (Fig. 2e). N-CHDM was notably better on NSCs proliferation on day 4 than S-CHDM. When each cell morphology was quantitatively analyzed, average cell area on all the substrates was quite similar to each other (Fig. 2f) but cell circularity on N-CHDM was significantly lower (Fig. 2 g).

**Fig. 3:**
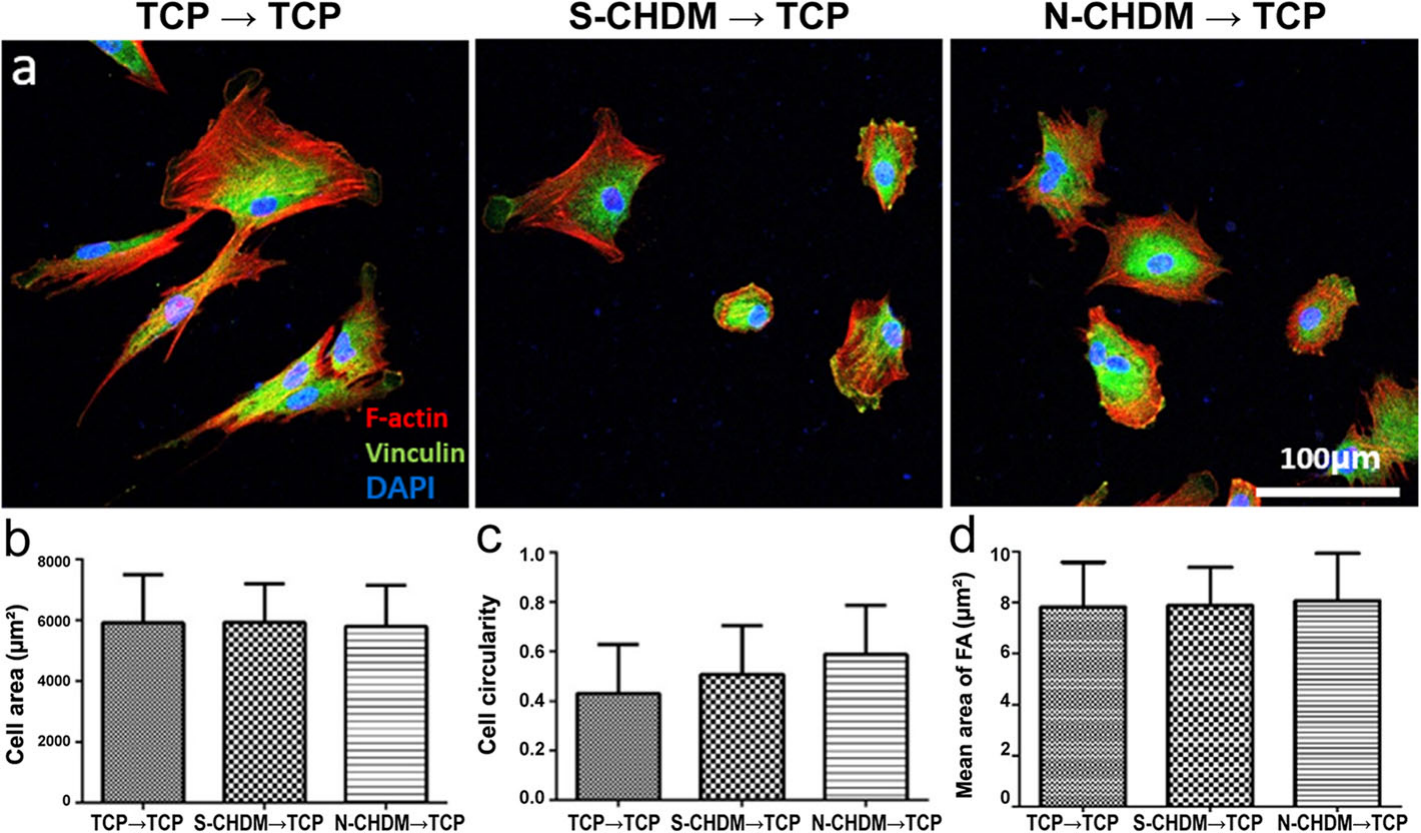
Observation of preconditioned NSC morphology on TCP. Preconditioning of NSCs on three different substrates for 7 days and re-plating them on TCP; **a** Appearance of cell morphology on TCP at 24 h: F-actin (red), DAPI (blue) and vinculin (green). The scale bar is 100 μm. Quantitative analysis of (**b**) cell area and (**c**) cell circularity, as determined by imageJ. Quantitative comparison of mean area of focal adhesion (FA) among the test groups (**d**)

**Fig. 4:**
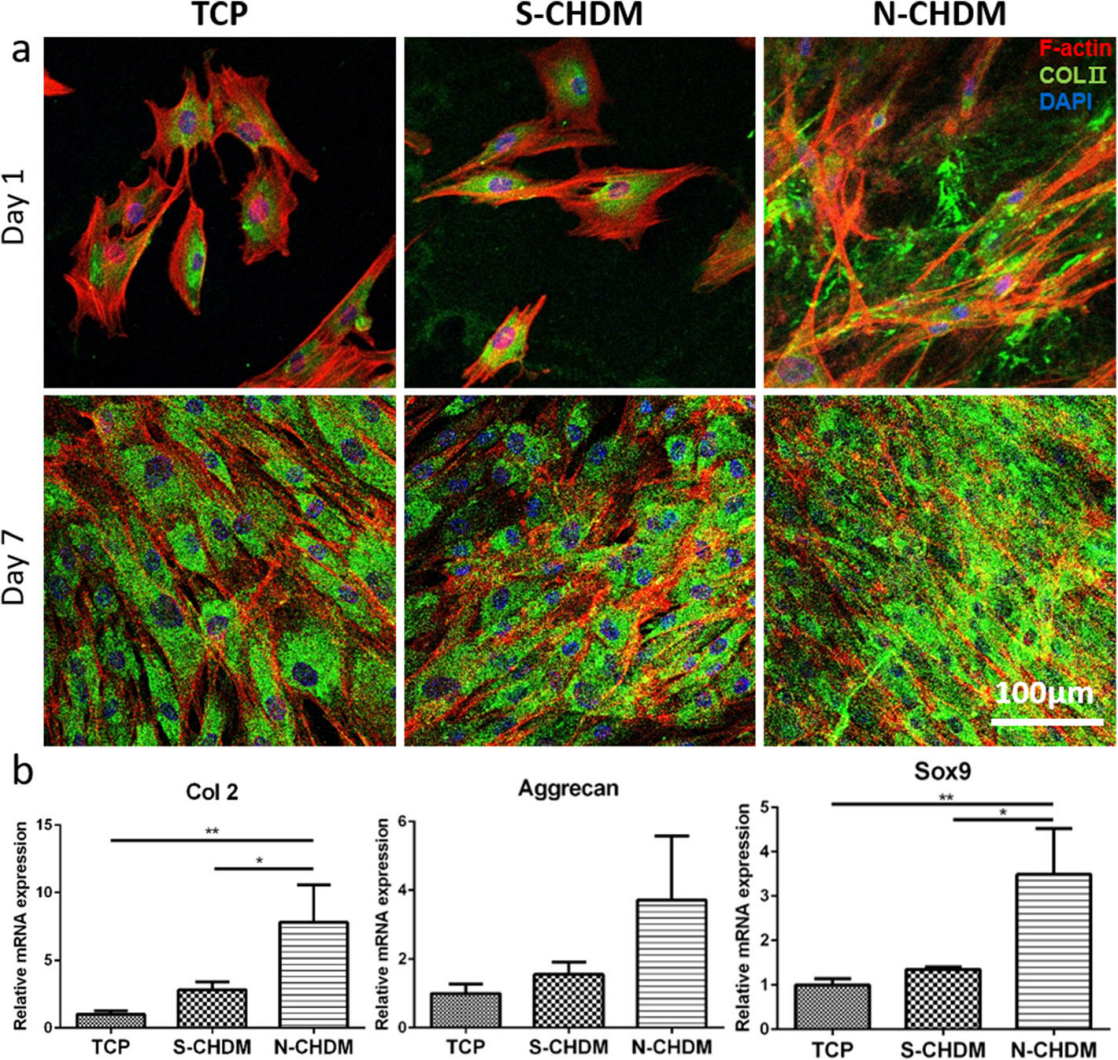
Chondrocyte differentiation of NSC on three different 2D substrates . NSCs are subject to cell differentiation on TCP, S-CHDM, and N-CHDM, respectively: **a** Representative images of F-actin (red) and COL II (green), as assessed via immunofluorescence, along with DAPI (blue) staining at 1 and 7 days. The scale bar is 100 μm. **b** Gene expression of Col2, Aggrecan, and Sox9, as examined via q-PCR on day 7. The expression levels are normalized to that of control (TCP) (*n* = 3, each group). Statistically significant difference as marked *p < 0.05 or **p < 0.01

In a separate study, we grew NSCs (P5) on three different substrates for 7 days, collected them and re-seeded on TCP, respectively. The results showed that the cell shape and focal adhesion on TCP was just similar to each other (Fig. 3a). It was notable however that the cell morphology was significantly different from the one appeared on the N-CHDM (Fig. 2b). Quantitatively there was little difference of cell area and circularity among the test groups (Fig. 3b and c). These results suggest that cell morphology of NSCs is highly affected by substrate microenvironment. In addition, the difference of mean area of focal adhesion (FA) was not significant among the test groups (Fig. 3d). Regarding chondrogenic differentiation of NSCs on each substrate, immunofluorescence of NSCs revealed that COL II was notable on N-CHDM at 1 day and more productive in the extracellular region at 7 day than TCP or S-CHDM (Fig. 4a). Quantitative analysis of chondrogenic markers (Col2, Aggrecan and Sox9) also presented advanced effect of N-CHDM in the maintenance of chondrogenic potential of NSCs (Fig. 4b). The difference of gene expression (Col2 and Sox9) was statistically significant between N-CHDM and the two counterparts.

### NSCs-mesh scaffold culture and chondrocyte differentiation ***in vitro***

**Fig. 5:**
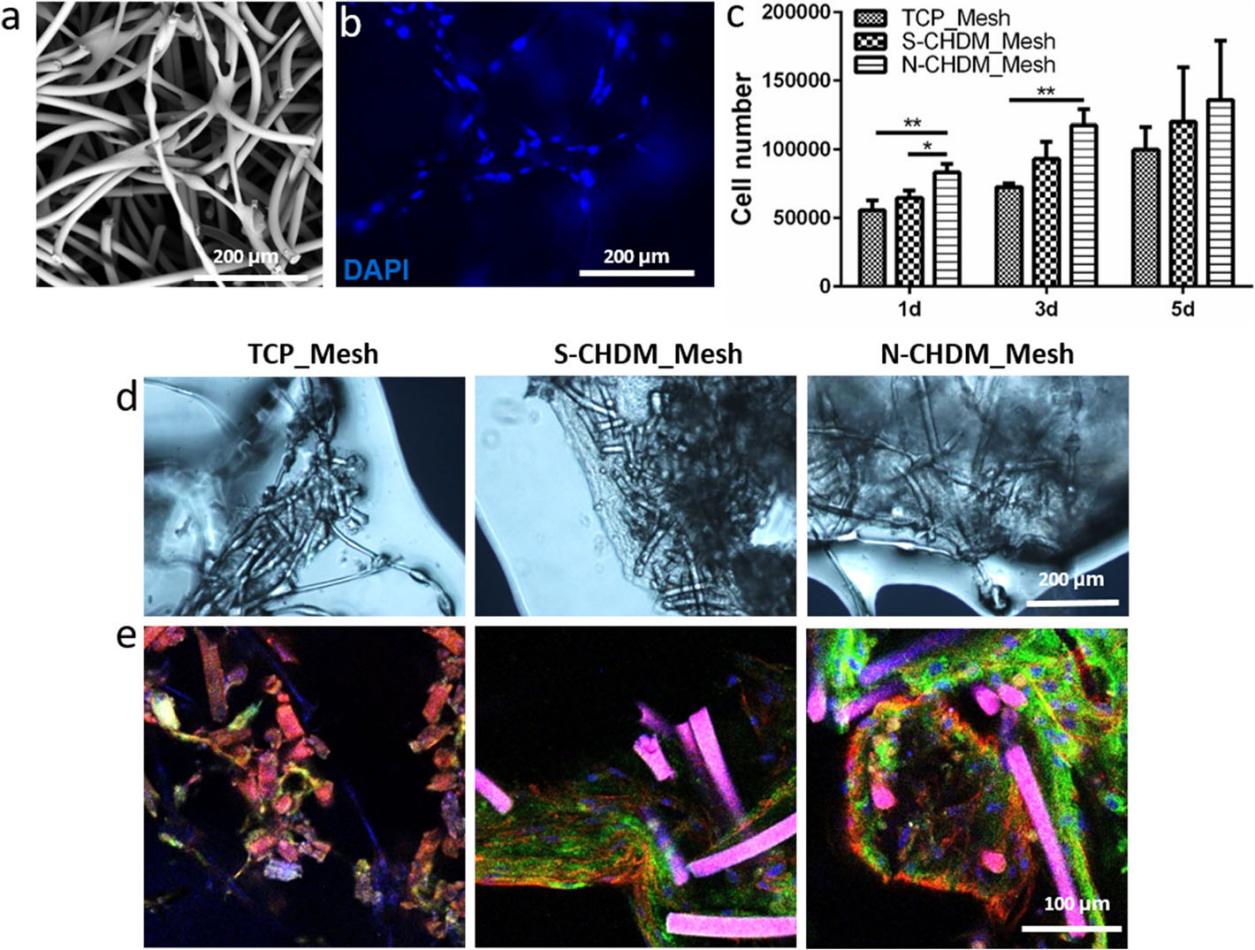
Preconditioned NSCs in the 3D mesh scaffold: Cell attachment, proliferation, and differentiation. **a** Appearance of the mesh scaffold itself via SEM and **b** observation of NSCs-seeded mesh scaffold at 1 day via DAPI staining. The scale bar is 200 μm. **c** NSC proliferation in each mesh scaffold at 1, 3, and 5 days as assessed via CCK-8 assay (*n* = 3, each group). TCP_Mesh, S-CHDM_Mesh, and N-CHDM_Mesh suggest the experimental groups loaded with NSCs preconditioned on different 2D substrates: TCP, S-CHDM, and N-CHDM, respectively before transfer to mesh scaffolds. **d** Appearance of the NSCs-mesh scaffolds at 3 weeks via phase contrast microscope. **e** Representative immunofluorescence images show the NSCs-derived, newly synthesized COL II (green) in the mesh scaffolds at 3 weeks, along with F-actin (red) and DAPI (blue) staining. The pink color indicates the mesh scaffold. Statistically significant difference as marked *p < 0.05 or **p < 0.01

To demonstrate the preconditioning effect of NSCs cultivated on three different microenvironments, they were collected from each substrate and transferred into a fibronectin-coated polymer scaffold (Fig. 5a) to examine NSCs proliferation and differentiation. We found the transplanted NSCs attached in the scaffold, as observed via DAPI staining (Fig. 5b). The number of NSCs gradually increased in the scaffolds and there was no significant difference on cell proliferation among the test groups (Fig. 5c). After 3 weeks of NSCs differentiation, we photographed the mesh scaffolds via optical microscope (Fig. 5d) and the immunofluorescence images showed that chondrocyte-specific matrix protein (COL II) was more productive with the NSCs preconditioned on the N-CHDM substrate as assessed via the intensity and distribution of fluorescence signal (green) (Fig. 5e).

**Fig. 6:**
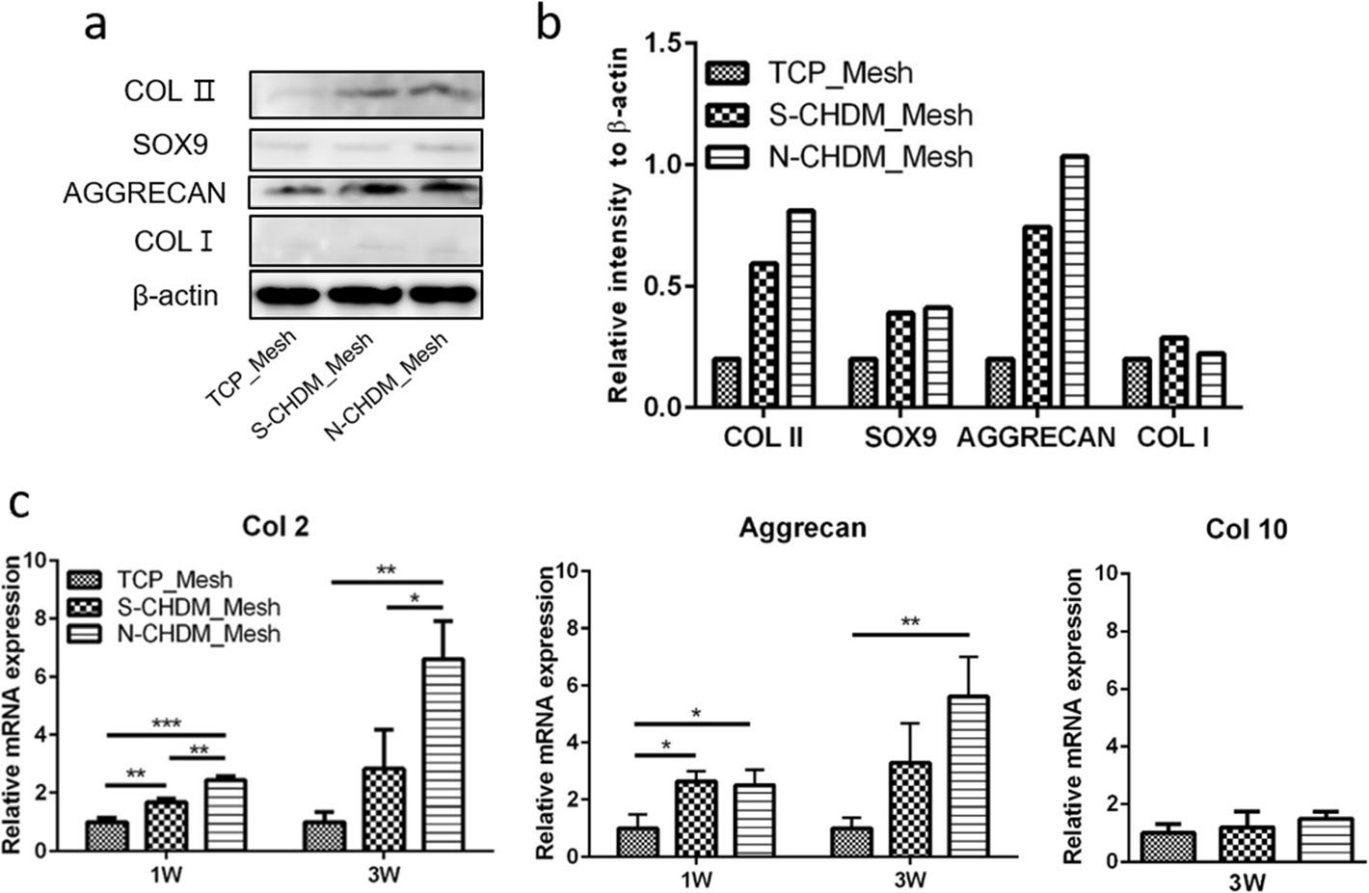
Preconditioned NSCs in the 3D mesh scaffold: Western blot and gene expression . **a** Examination of NSCs-derived macromolecule synthesis of COL II, SOX9, AGGRECAN and COL I using Western blot at 3 weeks: β-actin as a control. **b** Comparison of relative band intensity as assessed via imageJ. **c** Gene expression of Col2 and Aggrecan at 1 and 3 weeks, respectively and Col10 at 3 weeks. Statistically significant difference as marked *p < 0.05, **p < 0.01 or ***p < 0.001

Further analysis of Western blot demonstrates that the cartilage-specific COL II and aggrecan are more actively synthesized from those NSCs that were preconditioned on the N-CHDM and S-CHDM than on TCP (Fig. 6a and b). However the synthesis of SOX9 and COL I was rather weak for all the groups. It is notable that lower level of COL I is a positive signal for the maintenance of chondrocyte phenotype. We also found that the gene expression of chondrogenic markers (Col2, Aggrecan) largely increased with time, especially for N-CHDM group and showed statistically significant difference between TCP and N-CHDM at 1 and 3 weeks (Fig. 6c). In addition, the expression of Col10, an indicative of chondrocyte hypertrophy was barely different among the test groups at 3 week. Taken together, NSCs preconditioned on the N-CHDM are surely advantageous in encouraging the chondrogenic activity even in 3D environment than those preconditioned on TCP.

### NSCs-mesh scaffold transplantation ***in vivo***

**Fig. 7:**
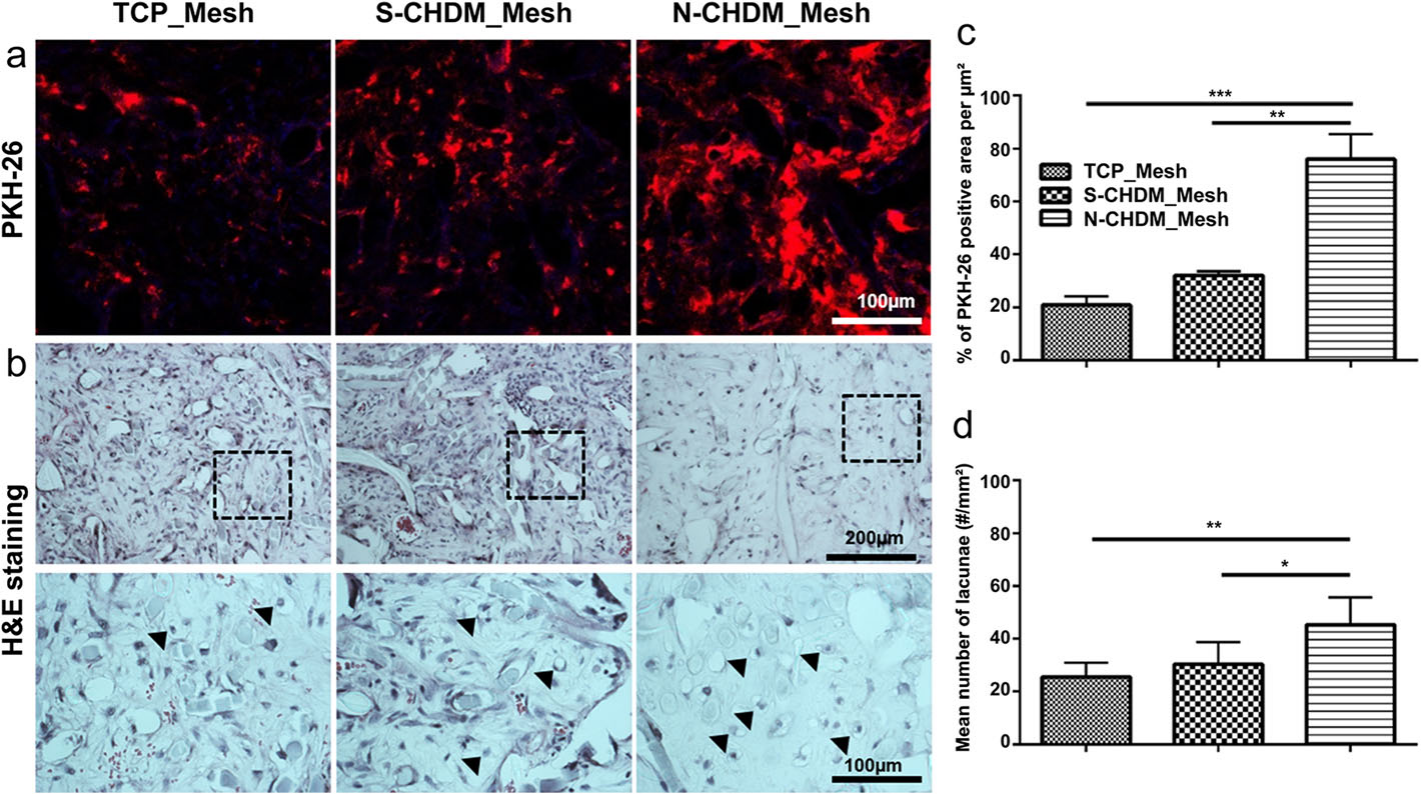
Investigation of the preconditioned NSCs retention and chondrocyte phenotype in the mesh scaffolds upon subcutaneous transplantation  in vivo. Once the preconditioned NSCs were pre-labelled using PKH-26 dye, they were loaded in the mesh scaffolds, then subcutaneously transplanted into nude mouse (n = 4, each group). **a** PKH-26 positive NSCs as confirmed via confocal microscope at 3 weeks post-transplantation. **b** Representative H&E stained images of NSCs in the mesh scaffolds. The black triangle indicates the NSCs with a lacunae structure. **c** Quantitative analysis of percentage of PKH-26 positive area per unit area (µm2) as determined via imageJ. **d** The mean number of NSCs with lacunae structure per unit area (mm2) is presented. Statistically significant difference as marked *p < 0.05, **p < 0.01 or ***p < 0.001

**Fig. 8:**
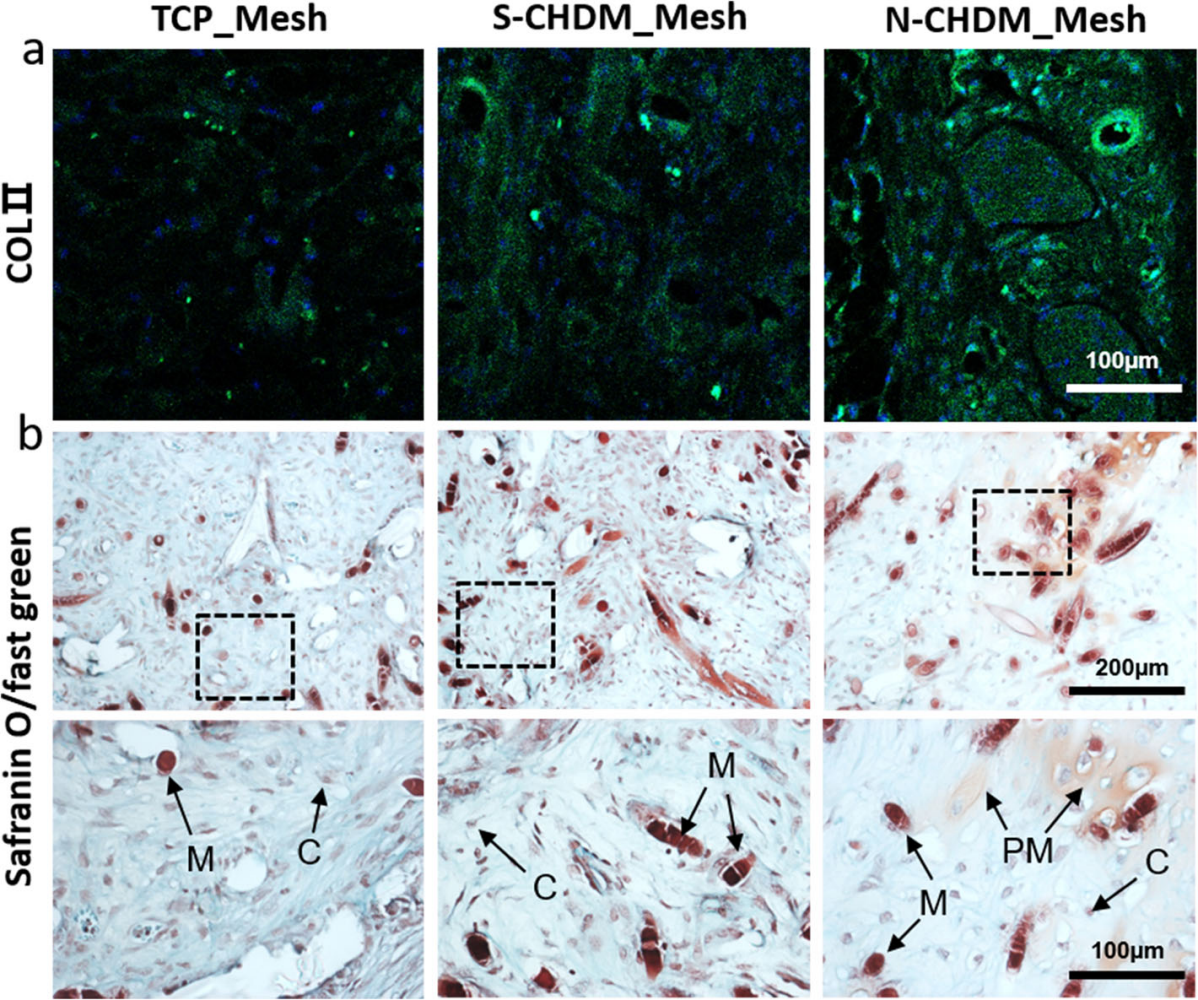
Chondrocyte differentiation of the preconditioned NSCs upon subcutaneous transplantation in vivo. Analysis of subcutaneously transplanted mesh scaffolds at 3 weeks: **a** Representative immunofluorescence images exhibit the positive signals of COL II (green) in the mesh scaffolds, along with DAPI (blue) staining. **b** The NSCs-derived, newly synthesized GAG is detected via Safranin O/fast green staining of mesh scaffolds. The boxed region is enlarged in a high resolution, where M is the mesh scaffold, PM is a pericellular matrix (GAG), and C is chondrocyte (NSC)

As for *in vivo* ectopic model, the pre-conditioned NSCs for 7 days on TCP, S-CHDM, and N-CHDM, respectively were subject to pre-labeling via PKH-26 dye. Those pre-labelled cells were then transferred into the mesh scaffolds and stabilized for 3 days before subcutaneous transplantation. After 3 weeks, we found that compared to TCP_Mesh and S-CHDM_Mesh, the PKH-26 positive signal in the scaffolds was significantly larger and more extensive with N-CHDM_Mesh, as assessed via PKH-26 cell tracker (Fig. 7a). Quantitative analysis also confirms a statistically significant difference between N-CHDM_Mesh and the other two groups (Fig. 7c). H&E staining identifies a lacunae structure that is unique for chondrocyte and an indicative of chondrocyte phenotype (black arrows in Fig. 7b). We noticed more chondrocytes with lacunae structure from the NSCs pre-conditioned on the N-CHDM, as supported by the number of chondrocyte lacunae in the given area (Fig. 7d). Additionally, the synthesis of COL II and another cartilage matrix, glycosaminoglycan (GAG) were more active and productive with the NSCs pre-conditioned on the N-CHDM, as assessed via immunofluorescence (Fig. 8a) and Safranin O staining (Fig. 8b). The GAG was detected as a peri-cellular matrix (PM).

## Discussion

Articular cartilage regeneration is highly demanded in clinic, because of increasing number of osteoarthritic patients [23]. Unfortunately the functional recovery of such cartilage is still a challenging task for both clinicians and researchers [24]. Among the numerous issues in cartilage regeneration, cell source would be a major interest [25]. Traditionally, primary chondrocytes harvested from articular knee cartilage have been utilized for autologous chondrocyte transplantation in clinics [26]. Human mesenchymal stem cell (hMSC) is a promising alternative to the primary chondrocytes, due mainly to some critical reservations, such as chondrocyte dedifferentiation and limited cell source [27]. hMSCs can be differentiated into chondrogenic cells and obtained from various human sources [28]. In this study, we examined NSCs as a cell source of cartilage tissue engineering, particularly focusing on the effect of NSCs preconditioning via NSCs-derived, decellularized ECM on chondrocyte differentiation *in vitro* and *in vivo*. Direct comparison of N- and S-CHDM clearly shows that N-CHDM provides better microenvironment for chondrogenic activity of NSCs on 2D substrate compared to S-CHDM. Moreover, as the NSCs preconditioned on N-CHDM were reloaded in polymer 3D mesh scaffolds, they could hold such chondrogenic potential in 3D environment as well.

Our findings may pose a crucial issue about the real face of ECM microenvironments that the NSCs interact with. In fact, N-CHDM retains significantly different ECM characteristics over S-CHDM. The surface topography is significantly different: rough, uneven surface of N-CHDM versus smooth, flat surface of S-CHDM (Fig. 1e and f). More specifically, N-CHDM shows the feature of fibrillar ECM structure but S-CHDM does not, because such distinct biophysical ECM property completely disappeared during the pepsin digestion process. Of course, we assume compositional variations between N- and S-CHDM can also be significant. Altered ECM compositions by enzymatic treatment may have contributed to reducing the chondrogenic potential of S-CHDM. Our results support that the NSCs preconditioned on the natural ECM microenvironment are more competitive in promoting chondrocyte differentiation under 2D as well as 3D condition. This implicates that natural ECM composition and ECM microstructure are crucial and should be reserved. While diverse signaling cues ECM delivers have never been directly compared, it sounds more reasonable that ECM composition alone may possess limited cues compared to those stemmed from natural ECM architecture and composition [29]. It is notable that N-CHDM composition is not limited to Col II and FN (Fig. 1). Rather there must be more diverse ECM constituents in the N-CHDM not fully evaluated in this study. In addition, regarding the mechanism of preconditioning effect, cellular and molecular mechanisms behind is currently unknown and thus warrant an in-depth study. We presume that some specific genes and/or proteins may be overexpressed with the cells preconditioned on the N-CHDM compared to the other substrates.

Meanwhile, poor cell survival rate after cell transplantation *in vivo* is one of the major hurdle in achieving a successful clinical outcome of cell therapy. In this sense, preconditioning of growing cells *in vitro* has been recognized a simple and practical strategy to enhance the cellular function and/or adaptability before cell transplantation. For example, the preconditioned MSCs using H_2_O_2_ improved MSCs proliferation and cell viability [30]. Interestingly, preconditioning of the MSCs *ex vivo* by inflammatory stimulus was found to be an adaptive strategy that made MSCs survive in the harsh environment *in vivo* and enhance their regulatory function of the local immune responses when injected [31]. Another study looked into the effect of low oxygen tension and confirmed its capability to encourage the regenerative potential of MSCs for cartilage repair [32]. Chondrogenic preconditioning using TGF-beta in culture medium also supported robust chondrogenesis of MSCs where they were encapsulated into self-assembled peptide hydrogel [33]. In addition, preconditioning MSCs via decellularized basement membrane like ECM resulted in better cell proliferation, chondrogenic potential, and pro-redifferentiation [34]. As mentioned, biochemical-based preconditioning has been predominant and cell preconditioning via a microenvironment control is a very promising approach. However how to standardize such environment must be a scientific and technical hurdle we need to pay great attention in the future.

It is also notable to mention the distinct NSCs morphology at 24 hr post-seeding on N-CHDM, compared to the one on TCP or S-CHDM (Fig. 2b-b’’). They show irregular morphology with highly stretched lamellipodia at the cell edge and exhibit significantly lower cell circularity. In general, higher cell circularity (close to 1.0) means more rounded cell shape, which is an indicative of cell phenotype of articular chondrocyte. Since the NSCs on TCP or S-CHDM showed significantly larger cell circularity (Fig. 2 g), chondrocyte differentiation should also have been active with the two substrates. As assessed via gene expression, immunofluorescence, and Western blot, however our results demonstrate that N-CHDM provides much better chondrogenic microenvironment for NSCs with lower cell circularity. Interestingly, once detached from N-CHDM and reseeded on TCP, those NSCs recover cell circularity as comparable to the ones detached from TCP and S-CHDM (Fig. 3). Since cell adhesion pattern is significantly different with NSCs attached on either smooth substrate (TCP and S-CHDM) or rough one (N-CHDM), the mechanism behind would rely on cell-substrate interactions that they can communicate bidirectinally. Such communications involve cell surface receptors, focal adhesion molecules, and specific proteins on signal transduction pathway [35]. We recognize that our work falls well short of discovering such communications and thus further in-depth study is warranted. In this study, we are just interested in the difference of soluble and natural ECM on cellular responses. There have been no such reports so far.

Cell source is always a critical issue in the field of cell therapy and tissue engineering. NSCs are one of the useful resources that are harvested from the patients in clinics. Previous study reported a spontaneous chondrogenic potential of NSCs, such as GAG synthesis, large amount of type 2 collagen, even when chondrogenic growth factors are unavailable [36]. A study of nasal septum cartilage showed no age variations in terms of hydration and collagen contents level compared to the knee cartilage [37]. This should be a strong advantage when NSCs are intended be administered for clinical application. Another interesting study showed that NSCs could maintain chondrogenic properties after extensive culture expansion of NSCs [20]. Moreover they noted that engineered catilage grafts prepared from nasal chondrocytes can be used clinically for the repair of articular cartilage defect in the knee after a first-in-human trial.

## Conclusions

In this study, we have demonstrated that natural ECM microenvironment (N-CHDM) would play a crucial role in enhancing NSCs proliferation and more importantly, promoting chondrocyte differentiation. The N-CHDM preconditioned NSCs on 2D and in 3D showed more advanced chondrogenic activity over those on S-CHDM or TCP. Even though the exact mechanism is unclear at this time on how N-CHDM can facilitate NSCs differentiation, our results suggest that N-CHDM may hold more diverse signaling cues, not just limited to ECM component. This study poses some implications regarding decellularized ECM engineering toward 3D bio-printing, organoids, and scaffold design.

## Data Availability

All data generated or analyzed in this study are included in this published article.
